# The Rise of Pluripotent Stem Cell-Derived Glia Models of Neuroinflammation

**DOI:** 10.3390/neurolint17010006

**Published:** 2025-01-13

**Authors:** Srishti Kala, Andrew G. Strutz, Moriah E. Katt

**Affiliations:** 1 Cancer Cell Biology Graduate Education Program, School of Medicine, West Virginia University Health Science Center, Morgantown, WV 26506, USA; sk00022@mix.wvu.edu; 2 Department of Microbiology, Immunology, and Cell Biology, School of Medicine, West Virginia University Health Science Center, Morgantown, WV 26506, USA; ags0004@mix.wvu.edu; 3 Department of Chemical and Biomedical Engineering, West Virginia University, Morgantown, WV 26506, USA; 4 Department of Neuroscience, School of Medicine, West Virginia University Health Science Center, Morgantown, WV 26506, USA

**Keywords:** neuroinflammation, human pluripotent stem cells, astrocyte, microglia, oligodendrocyte precursor cell, pericyte, brain microvascular endothelial cell, in vitro modeling

## Abstract

Neuroinflammation is a blanket term that describes the body’s complex inflammatory response in the central nervous system (CNS). It encompasses a phenotype shift to a proinflammatory state, the release of cytokines, the recruitment of peripheral immune cells, and a wide variety of other processes. Neuroinflammation has been implicated in nearly every major CNS disease ranging from Alzheimer’s disease to brain cancer. Understanding and modeling neuroinflammation is critical for the identification of novel therapeutic targets in the treatment of CNS diseases. Unfortunately, the translation of findings from non-human models has left much to be desired. This review systematically discusses the role of human pluripotent stem cell (hPSC)-derived glia and supporting cells within the CNS, including astrocytes, microglia, oligodendrocyte precursor cells, pericytes, and endothelial cells, to describe the state of the field and hope for future discoveries. hPSC-derived cells offer an expanded potential to study the pathobiology of neuroinflammation and immunomodulatory cascades that impact disease progression. While much progress has been made in the development of models, there is much left to explore in the application of these models to understand the complex inflammatory response in the CNS.

## 1. Introduction

Our understanding of neuroinflammatory processes often relies heavily on data obtained from heterologous cell models, primarily those derived from non-human organisms. This reliance introduces a crucial caveat: species-specific disparities exist in the cellular and molecular mechanisms governing neuroinflammation. These discrepancies can significantly impede the translation of preclinical findings to clinical application [1,2]. For example, human astrocytes are larger and more complex, display greater heterogeneity, and have a different astrocyte–neuron ratio than murine astrocytes [3]. In fact, two of the four categories of GFAP+ astrocytes, varicose projection [4] and interlaminar astrocytes [5], are human-specific and are not easily studied in rodent models. Genome-wide association studies (GWASs) and transcriptomic studies have revealed that genes relevant to neurodegenerative disease are differentially expressed in rodents and humans, including APOE [6,7]. Mice with human APOE4 microglia show increased HIF-1α expression, and disease-phenotypic glycolysis [8]. Similarly, human brain pericytes and endothelial cells also demonstrate distinct phenotypes [9,10].

Human primary cell types relevant to neuroimmune mechanisms, such as brain microvascular endothelial cells, neurons, microglia, and astrocytes, are difficult to obtain in sufficient quantity to allow reproducibility. While the primary culture of CNS cells is an essential tool for studying neuroinflammation in vitro [11], these cell types often have limited to non-existent expansion capabilities [12], as mature CNS cells are largely senescent [13]. An exception includes some primary human microglia that have been successfully maintained in high-pass culture in the long term with M-CSF [14,15]. Obtaining a human primary CNS culture requires time-sensitive post-mortem collection and carries a donor’s unknown life and epigenetic history while providing a finite quantity of cells. Both human and murine cells lose essential phenotypes when examined ex vivo in extended culture, possibly due to the loss of environmental cues [16]. Immortalized and proliferative human CNS lines have unique phenotypes that partially recapitulate that seen in vivo and may not respond to stimuli appropriately, though they can be valuable tools for exploratory studies [17].

Human pluripotent stem cells (hPSCs) allow scientists to address some of these challenges; hPSCs, particularly human induced pluripotent stem cells (hiPSCs), have been differentiated into cells with distinct and relevant phenotypes for neuroinflammation research. These include neuron-, brain-endothelial-, and pericyte-like cells and glial cells such as astrocyte-, oligodendrocyte-, and microglia-like cells [18,19,20]. hPSCs are readily expandable, enabling the production of a nearly infinite quantity of cells; the resultant derived cells express many of the essential markers and phenotypes seen in vivo. These hPSC-derived cells can then be incorporated into a wide variety of in vitro methodologies. hPSC-derived cell types within neuroinflammation research have been used in monoculture and polyculture in 2D and 3D environments, ranging from simple monocultures [21] to transwells [22], spheroids [23], organoids [24], and microfluidics [25].

hPSC-derived CNS cell types allow novel methodologies for conducting basic research with bench-to-bedside implications. The potential applications in personalized medicine are an additional advantage of hiPSCs, as hiPSCs can be created for individuals with genetic risk factors to determine the specific biology and test potential therapeutic strategies. This can give researchers insight into numerous CNS diseases that carry disease-causing genomic risk factors. This can be seen when looking at brain microvascular endothelial-like cells in the context of multiple sclerosis (MS) [26] or in microglia-like cells impaired during phagocytosis in Parkinson’s Disease (PD) [27]. Further examples will be discussed throughout this review.

**Figure 1:**
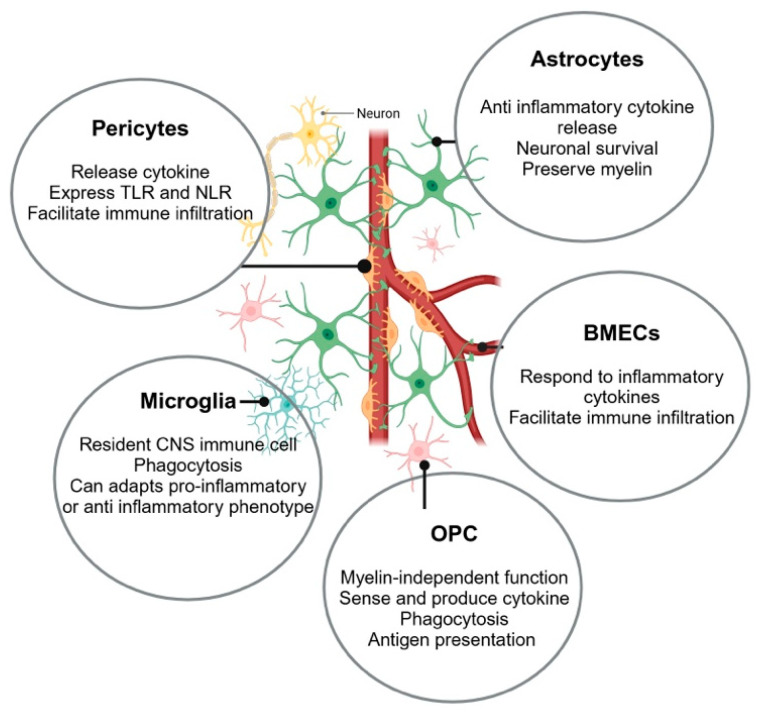
Neuroinflammatory response at the NVU. Summary of the impact of the glial support cells in the neural vascular unit (NVU) and their role in neuroinflammation. The NVU plays a critical role in neuroinflammation. Brain microvascular endothelial cells (BMECs) forming the BBB are essential for maintaining brain homeostasis. However, under inflammatory conditions, disruption of tight junctions and adherens junctions in the BBB allows for infiltration of immune cells. Microglia, oligodendrocyte precursor cells (OPCs), pericytes, and astrocytes contribute to this inflammatory response by releasing proinflammatory cytokines, chemokines, and reactive oxygen species. These cells interact with each other, amplifying the inflammatory response and leading to neuronal damage and death. The extracellular matrix (ECM) provides a structural framework for these interactions and facilitates the spread of inflammatory signals throughout the NVU.

The most extensive use of hPSC-derived cell types of the CNS has been found in studying neuron-like cells [28,29,30]. The study of hPSC-derived glial cells within the CNS is in its comparative infancy. There have been significant advancements in understanding neuroinflammatory mechanisms because of these new techniques, and more are still to come. Many cells within the CNS contribute to neuroinflammation. This review will focus on microglia, astrocytes, oligodendrocyte precursor cells, endothelial cells, and pericytes (Figure 1). Here, we will review key findings from recent papers highlighting these CNS cells and their role in neuroinflammation.

## 2. Microglia

Microglia are the tissue-resident macrophages and primary immune cells of the CNS [31]. Microglia are macrophages that migrate from the yolk sac to colonize the early CNS during embryogenesis, prior to neurogenesis, in contrast to their cousins, the peripheral myeloid-precursor-derived macrophages [32]. The roles of microglia are diverse and expanding including innate immune function and adaptive immune function, as well as roles in homeostasis and neurogenesis [33,34,35]. Microglia have been shown to phagocytize bacteria [36], damaged cells [37], and protein aggregates such as amyloid-beta (Aβ) [38] and alpha-synuclein (aS) [39]. In the context of inflammation and blood–brain barrier (BBB) hyperpermeability, microglia can upregulate both MHC I and II and present and migrate with T cells to peripheral-CNS-serving lymph nodes [40,41]. Transcriptomic and phenotypic heterogeneity reflects a multilayered, plastic continuum [42].

hiPSC-derived microglia-like cells (hiMGs) are increasingly being used to model immune parenchymal interactions in the context of neuroinflammation and neurodegeneration [43,44]. There is demand for human in vitro modeling of the CNS to complement existing human or rodent in vivo and ex vivo systems [45,46,47]. Including microglia in the modeling of CNS disease has elucidated critical pathomechanisms due to the specificity that requires in vitro methodology that otherwise cannot be studied using other conventional approaches such as slice cultures [48]. The use of hPSC-derived cells with microglial phenotypes may address this demand to improve basic science understanding and develop novel therapies for diseases of neuroinflammation.

### 2.1. Differentiation Concepts

Different approaches to generating hiMGs have been attempted with various phenotypes being characterized [49]. Markers used to identify hiMG in culture often include microglia-specific TMEM119 [50], P2RY12 [51], and TREM2 [52] with co-expression of less specific monocyte/macrophage CD11b [53] and leukocyte common antigen CD45 [53]. hiMGs have exhibited functions indicative of in vivo microglia including phenotypic plasticity to damage- and pathogen-associated molecular patterns (DAMPs/PAMPs), the production of cytokines/chemokines, and phagocytotic ability [49].

There are two main classes of hiMG differentiations based on the progenitor stage that they proceed through. Early attempts included proceeding through a hematopoietic-like stem cell lineage, resulting in peripheral macrophage-like cells with microglial phenotypes [54,55]. More recent protocols attempt to partially mimic the unique yolk-sac erythromyeloid progeny, which is more indicative of development in vivo [56]. Efforts to increase efficiency and decrease the duration of these differentiations have been made by encoding transcription factors highly expressed in microglia. Dräger et al. engineered an hiPSC line to transiently express six transcription factors (Hematopoietic Transcription Factor PU.1, MAF BZIP Transcription Factor B, CCAAT Enhancer Binding Protein Alpha, CCAAT Enhancer Binding Protein Beta, Interferon Regulatory Factor 5, and Interferon Regulatory Factor 8) to shorten the differentiation to eight days [57]. These transcription factor-induced microglia also exhibit classical microglia behavior including response to PAMPs, synaptosome formation, and cytokine excretion [57].

### 2.2. Neuroinflammatory Insights

While microglia are the resident immune cells of the brain, studies that have applied the hiMG to study neuroinflammatory pathways and mechanisms in disease are still relatively few in number. Mechanisms of neuroinflammation are often shared across disease states, and the current literature on microglia elucidates common disease non-specific neuroimmune pathways. These are summarized in Table 1.

hiMGs have demonstrated similar transcriptomes and many similar functions to primary human microglia [38,58]. Microglia are known to have many important characteristics of their peripheral macrophage counterparts while having distinct roles inside the brain. In response to ADP, hiMGs increased Ca^2+^, consistent with human primary microglia, indicating that cells have functional P2RY12, a microglia-specific purinergic receptor involved in neuroinflammation [58].

hPSC microglia used to model specific disease states have been most utilized in Alzheimer’s disease (AD) research. Microglia are suspected to help clear up Aβ aggregates, and hiMG are able to phagocytize Aβ aggregates and release intracellular Ca^2+^ in response to environmental ADP, demonstrating their potential role in AD pathobiology [38]. TREM2 is a known risk factor in AD and is known to be a key regulating protein in microglia. Using a TREM2 knockout (KO), hiMGs show increased inflammatory phenotypes, with increased intracellular Ca^2+^ release upon ATP/ADP stimulation and a shift toward a complement C5a activation phenotype [63]. Previous work with TREM missense mutations revealed a less severe proinflammatory state [64]. Similarly, hiMGs from APOE4 hiPSCs demonstrate increased proinflammatory phenotypes and impaired phagocytosis of Aβ and show increased expression of genes associated with a proinflammatory state [60]. Using APOE4 variants and gene-edited controls, TCW et al. identified a unique transcriptional profile shift in hiMGs that is reflective of human disease pathology and shows clear species-specific differences, again underlying the critical importance of human-based models [59].

Outside of AD, neuroinflammatory studies incorporating hiMG are less common, Haenseler et al. showed that hiPSCs–macrophages with Parkinson’s Disease (PD)-associated genes had increased expression of alpha-synuclein (aS) resulting in M1-like phenotypes and distributed phagocytic ability [65]. Transcriptomics of iPSC microglia derived from patients with PD show upregulation of IL-1β from the NLRP3 inflammasome in response to inflammatory molecules compared to negative controls [27].

Using iPSC microglia to study mechanisms specific to other diseases involving neuroinflammation where microglia are hypothesized to play a prominent role in vivo is an emerging topic and has been underutilized in the context of ischemic stroke, MS, amyotrophic lateral sclerosis (ALS), traumatic brain injury (TBI), frontotemporal dementia (FTD), and chronic infections of the CNS.

## 3. Astrocytes

Astrocytes are the most abundant, pleiotropic, and homeostatic cells in the brain with various roles ranging from development, metabolism, blood–brain barrier (BBB) maintenance, waste and monoamine clearance, and immune functions in the CNS [66]. Astrocytes are a heterogeneous cell type with a diverse phenotypic continuum [67]. The role of astrocytes in neuroinflammation has been reviewed elsewhere [68,69,70,71]. Like macrophages and other traditional immune cells, astrocytes can be dichotomized from a homeostatic state (A_0_-like) to proinflammatory (A_1_-like) and anti-inflammatory (A_2_-like) states during disease states like neurodegeneration and cancer [68,72]. Paracrine astrocyte–other glia interactions are becoming increasingly examined during neuroinflammation, with this being particularly instigated by microglia [72]. Here, we focus on the neuroinflammatory and immune regulatory roles that astrocytes, in the context of hPSC astrocytes, have played in the enhanced understanding of their role within the brain; these findings are summarized in Table 2.

### 3.1. Differentiation Concepts

hPSC astrocyte-like cell (hiA) differentiations are relatively numerous and robust in comparison to other hPSC glia differentiations. This is likely due to their neural precursor/radial glia lineage as astrocytes in vivo share progenitors to neurons, one of the most well-established and utilized hPSC-derived cell types historically [73]. Early differentiations identified that hiAs are often reactive and immature immediately following differentiation and require extended culture in FGF to transition to a more quiescent mature phenotype [74]. Regarding FGF, more recent strategies include BMP inhibition and subsequent addition of other neurotrophic factors including LIF and CNTF that have resulted in successful GFAP+ and GLAST+ (hiA) differentiations [75,76,77]. Principal component analysis reveals that while hiA’s reactive inflammatory phenotype shows some similarity to primary astrocytes, there is still some ground that needs to be covered [78]. CD49f was identified as a novel astrocyte surface marker using hiA, it correlates with AQP4 and GFAP expression, and it is present in vivo [79]. In addition to being able to respond to inflammatory cytokines and upregulate GFAP, hiAs present a variety of functional phenotypes that would be expected in vivo. hiAs, in response to inflammatory stimuli, produce cytokines [17,76], phagocytize [80], including Aβ [81], uptake glutamate [21], and produce complements [82].

Characterization and identification of astrocytes is difficult as there is no one established pan-astrocyte marker/phenotype. Astrocytes are a highly heterogeneous cell population, so it is difficult to determine appropriate markers. Protein and gene level characterization are common; there is an increase in functional characterization including cholesterol synthesis and glutamate uptake [83]. Current differentiations have not been able to capture the diversity of astrocyte phenotypes seen in vivo. It may be that organoids could hold the answer to the differentiation of the wide variety of subtypes of astrocytes. Current work with cerebral organoids indicates that methamphetamine may cause neuroinflammation through an astrogliosis-mediated pathway [84]. Current efforts with hiA are largely centered around the transition to A_1_ from A_2_ as well as changes in different disease phenotypes. hiAs show robust inflammatory responses to treatment with TNFα with phosphorylated NF-κB; IL-1β also produces an inflammatory response but it is a less dramatic response [85]. Variability exists between hiA differentiations of the same hPSC line for the purposes of testing hypotheses comparable to working with animals and human primary cells. Mulica et al. (2023) characterized two hiA differentiation protocols, with and without serum, and compared them against adult human primary astrocytes via morphology and RNA sequencing [77]. They found that their serum-free differentiation protocol resulted in more mature phenotypes while the serum containing hiAs had a higher yield [77]. This highlights the need for appropriate differentiation protocol selection that would best correspond to the hypothesis being tested. Complements, a collection of soluble and membrane-bound proteins commonly attributed to the innate immune system, have increasingly pleiotropic functions in the CNS. A primary function includes complement-dependent-glia-mediated synaptic pruning during development, health, and disease [86]. Neuroinflammatory diseases implicate abnormal complement expression or function. A few of these diseases include multiple sclerosis (MS) [87], AD [88], and long COVID [89]. While neurons and diverse glia produce complements, astrocytes are the major producers in the CNS [90,91]. Complement-focused therapeutics for diseases of neuroinflammation are currently being investigated; however, glia–glia and glia–neuron-dependent interactions further complicate our understanding of complements in the brain [92]. hiAs provide accessibility to study complement in select human CNS-like cells in vitro [79,82,93,94].

### 3.2. Neuroinflammatory Insights

While in its early stages, hiAs have been used broadly in the modeling of neurogenerative diseases and have been reviewed previously [94]. hiAs are relevant in investigating diseases of demyelination, like multiple sclerosis (MS) and neuromyelitis optica (NMO). Key findings are summarized in Table 2. hiAs derived from patients with MS have differential responses to inflammatory cytokines but have indistinguishable gene profiles when inactive [76]. Taking this further, Kerkering et al. (2023) derived hiA and hiPSC neurons from patients with benign vs. progressive MS under co-culture with MS-associated cytokines [93]. The hiAs from the benign MS group expressed less inflammatory differences in growth factors and single-cell sequencing which was recapitulated in their neuron axons in co-culture [93]. Using hiAs, Cho et al. (2021) elucidated novel organelle-level changes during NMO, a demyelinating disease of the optic nerve, which was previously infeasible to conduct research using in vivo systems [95]. While aquaporin 4 (AQP4) autoantibodies are already established as the etiological cause of NMO, how the disruption of intracellular and organelle processes contributes to damage to the optic nerve is not clear. This lab-cultured hiA with NMO-patient serum and induced structural and functional changes to multiple organelles compared to negative controls [95]. These provide demyelinating-disease examples in which hiA research highlights potential targets for disease-modifying therapeutics that were previously unknown from other model systems.

**Table 2:** Use of hiA to study neuroinflammation in neurodegeneration.

Disease	Key Findings	Source
NMO	AQP4 autoantibodies from NMO patients induce detrimental metabolic and organelle changes in hiAs	[95]
AD	Transcriptomics of hiAs with APOE4 variants and KO reveal species-specific role of astrocytes in cholesterol metabolism in AD	[59]
MS	Transcriptomics and culture of HiAs derived from patients with different types of MS differential cytokine production and JAK-STAT activation hiA-dependent degradation of hiPSC neurons varies in MS subtypes	[93]
MS	Transcriptomic and in vitro analyses of hiAs under MS-associated cytokines show select differential gene regulation and combinatorial effect of specific cytokines	[76]
General inflammation	Transcriptomic and surface analysis of hiAs identified CD49f as a novel marker for A_1_-like astrocytes	[79]
General inflammation	Identified novel role of hiA-derived alpha 1-antichymotrypsin in inflammatory brain microvascular endothelial barrier dysfunction	[96]
AD	hiAs contribute to clearance and aggregation of Aβ	[97]
AD	Mid-throughput silencing of suspected genes implicated in AD using hiAs and hiPSC neurons	[98]
TDP-43 dementia	hiAs with TDP-43 mutations show higher expression of cytoplasmic TDP-43 and increased cell death	[99]

hiAs also have advantages for investigating neuroinflammatory diseases that are mechanical, e.g., stroke and traumatic brain injuries (TBIs), to supplement other model systems. Barbar et al. (2020) identified CD49f, i.e., integrin α6 as a novel biomarker for the use of sorting and identifying hiAs [79]. During culturing with proinflammatory molecules also used to model stroke in vitro (TNFα, IL-1α, C1q) [100], the hiAs had functional deficits in phagocytosis and monoamine clearance and released soluble factors detrimental to neurons across species [79]. Kim et al. (2022) identified an astrocyte glycoprotein, alpha 1-antichymotrypsin, as an inflammatory mediator during a model of brain vascular inflammation utilizing an hiA-hiBMEC co-culture [96]. They identified an hiA-dependent hiBMEC reduction in barrier phenotype under the same proinflammatory molecules used to model stroke [96]. Silencing the gene that encodes for alpha 1-antichymotrypsin in hiAs rescued homeostatic-level expression of VCAM-1 in hPSC-derived brain microvascular endothelial-like cells compared to negative controls and other proinflammatory genes. HiAs are starting to be used in TBI models. Lai et al. (2024) generated hiPSC-derived brain organoids which contain both neuron- and astrocyte-like cells [101]. At different timepoints in their culture, they modeled TBI via high-intensity focused ultrasound and observed a chronic increase in the expression of GFAP which is a well-characterized phenotype of TBI in vivo [101,102]. Together these examples display that hiAs are an emerging yet relevant model for studying mechanical diseases of neuroinflammation.

Astrocytes have been implicated in dementias and neurodegenerative diseases broadly [103] but are often examined in AD [104,105,106]. Bassil et al. (2021) generated hiAs along with hPSC neurons and microglia to further characterize hPSC-derived cells for modeling Alzheimer’s disease [97]. In response to exogenous amyloid-β (sAβ42s), their hiAs upregulated GFAP and stained positive for bound Aβ in both mono- and polycultures [97]. Although Bassil et al. recapitulated known in vivo phenotypes of AD with their hiAs, another approach is to examine previously suspected genes from datasets at a cell-specific, mid-to-high throughput level. Sullivan et al. 2019 performed systematic, high-throughput silencing of previously highlighted genes suspected to be implicated in AD in both hiAs and hPSC neurons [98]. They identified genes that have novel effects on the production and extracellular availability of Aβ40 and 42 in hiAs compared to hPSC neurons, solidifying the idea that astrocytes and hiAs are relevant cells and model systems for AD research.

## 4. Oligodendrocyte Precursor Cells (OPCs)

Oligodendrocyte precursor cells (OPCs), also known as NG2 glia, are another class of glia that mediate neuroinflammation and are increasingly being investigated [107,108,109]. OPCs are multipotent cells that give rise to oligodendrocytes, but humans also maintain a persistent self-renewing population of OPCs throughout adulthood [110]. OPCs are one of the most proliferative cell types in the adult human brain [111]. Oligodendrocytes are the primary myelin-producing cells within the brain; they ensheathe axons facilitating efficient action potentials, both during development and adulthood [112]. OPCs are highly studied in demyelinating diseases [113] as they migrate to demyelinating lesions and glial scars [114] where they further differentiate into oligodendrocytes and remyelinate to some degree. Despite the clear potential of delivering OPCs as a therapeutic strategy to increase remyelination, there are significant hurdles to inducing OPC differentiation as a therapeutic strategy [113,115].

The role and function of OPCs have primarily been thought to be maturation into oligodendrocytes and the subsequent formation of myelin; however, OPCs carry out distinct essential functions independent of oligodendrocyte maturation [116]. OPCs are understood to be increasingly pleiotropic, with neuroinflammation mediating functions that overlap with other glia. These immune cell-like functions, in response to neuroinflammation, include increased proliferation/migration [117], sensing and producing cytokines/chemokines [118], phagocytosis of damage and pathogen molecular patterns [119,120], and upregulation of MHCII for antigen presentation to CD8+ T cells [121] and contribute to ECM remodeling [122] and glial scar formation [123].

OPCs make up a small number of total cells in the brain, with comparatively higher amounts in white matter [112]. There are established species-specific differences between rodent and human OPCs; however, the extent of these differences has not been fully characterized. Single-cell transcriptomics suggest that there are human OPCs that express hundreds of non-orthologous genes when compared to rodents [124]. Maintenance and expansion of OPCs may have species-specific differences. Huang et al. examined human embryo OPC proliferation in the cortex; daughter and granddaughter OPC divide symmetrically and then self-repel, antithetical to radial glia expansion [111]. Like other glia, human OPCs are larger and have more heterogeneity in their expression function compared to mice [125].

Primary human OPCs are few in quantity and difficult to obtain, with human embryonic OPCs functionally distinct from adult OPCs [126]. OPC species-specific differences in gene expression, development, and function are significant barriers to discovering the role of OPCs in CNS pathology [111,124]. The use of hPSC-derived OPCs (hiOPCs) satisfies a niche required for further translational research related to OPCs. Despite the relative youth of hiOPCs and limited options for differentiations and commercially available cells, this is a growing space in the literature with increasing relevance in neuroinflammation. A summary of key findings can be found in Table 3.

### 4.1. Differentiation Concepts

hiOPC differentiation strategies either utilize a cocktail of growth factors or transfection and activation transcription factors to mimic developmental cues. In vivo, OPCs are derived from radial glial cells, the same progenitors as astrocytes and neurons. Therefore, differentiation strategies often begin similarly to other radial glia-derived cells, first generating a neural precursor neuroectoderm-like cell (NPC) using growth factors and supplements such as basic fibroblast growth factors N2 and B27 [127]. A major drawback of utilizing growth factors to mimic development is the duration of the differentiation; hiOPC protocols are amongst the longest differentiations. Differentiation strategies for hiOPCs using cocktails of growth factors can take around 6 months [128]. Transfection with key transcription factors has the benefit of producing hiOPCs in just a few weeks. Xu et al. (2022) have been able to generate hiOPCs in just 21 days from neuroectoderm-like cells using OLIG2-mRNA to activate SRY-box transcription factor 10 (SOX10) promoting hiOPC lineage [116].

### 4.2. Neuroinflammatory Insights

Like other glia, OPCs sense and secrete cytokines [129], and can perform antigen presentation in in vitro [130] and in vivo [121]. Due to the inflammatory nature of demyelinating diseases and the prominent role of oligodendrocytes in remyelination, much of OPC research has historically been focused on demyelinating diseases like multiple sclerosis, acute disseminated encephalomyelitis, and neuromyelitis optica (NMO) [131,132,133]. However, there is a more recent shift toward the myelin-independent immune-modulatory roles of OPCs in neuroinflammatory and neurodegenerative diseases more broadly [107,134,135,136]. Key results are summarized in Table 3.

**Table 3:** Use of hiOPCs to study neuroinflammation/neurodegeneration.

Disease	hiOPC Findings	Source
AD	hiAs with CLU deletion release proinflammatory cytokines/chemokines resulting in decreased hiOPC proliferation and basic myelin protein production	[137]
AD	hiOLs, not hiOPCs, produce higher levels of Aꞵ_40_	[138]
Secondary progressive MS	hiOPCs have diminished migratory phenotypes and secretomes	[128]

Demyelinating diseases account for the majority of hiOPC research; transplantation of hiOPCs is being investigated as a therapeutic strategy, with less emphasis on hiOPCs as a basic or translational research tool [139]. Generally, demyelinating diseases are etiologically driven by the generation of autoantibodies for myelin-associated proteins and subsequent cell-mediated destruction of oligodendrocytes [140]. Lopez-Caraballo et al. generated hiOPCs from patients with secondary progressive MS; the resultant cells had an attenuated migration ability in vitro compared to non-MS controls [128]. Comparison of secretomes between experimental groups displayed downregulation in proteins associated with proliferation, projection, and ECM interaction [128].

Elucidating the role of OPC in traditionally “non-demyelinating” neurodegenerative diseases broadly is a growing niche in the neuroinflammation space [141]. hiOPCs have been studied in co-culture with hiAs in an AD model looking at the effect of the clusterin (CLU) genetic risk factor for AD. CLU results in a more proinflammatory hiA phenotype, resulting in reduced proliferation of hiOPC and reduced basic myelin protein production [137]. This suggests that modulating the immune signaling of astrocytes may provide a strategy for improving the efficacy of hiOPC transplants. Rajani et al. (2024) generated a panel of iPSC-derived glia from patients with familial Alzheimer’s disease; hiMGs, hiAs, hiOPCs, and oligodendrocyte-like cells (hiOLs) were used to compare Aꞵ_40_ protection, and it was found that hiOLs derived from hiOPCs produced significantly more Aꞵ_40_ than any of the other cell types tested [138]. Basic and applied research with hiOPCs and hiOLs are required to isolate these cells without other cell interactions and species-specific differences.

The use of hiOPC in neuroinflammation research outside of transplantation for demyelinating diseases is a relatively innovative approach; as OPCs become increasingly implicated in dementias [142,143], stroke [144], and traumatic brain injury [145], there is a clear need to study their role in pathobiology. The hPSC-derived cells, which circumnavigate some of the challenges that traditionally come with researching OPCs, such as sourcing, quantity, time, and specifies-specific differences, offer a valuable tool in unveiling the role of OPCs in neuroinflammation across a wide variety of CNS conditions.

## 5. Brain Microvascular Endothelial Cells

Brain microvascular endothelial cells (BMECs) are the gatekeepers of the brain, controlling transport into and out of the brain; this strict control is needed to maintain brain homeostasis while keeping pace with the high metabolic demands of the brain [146]. BMECs are often seen as having an indirect role in neuroinflammation, acting instead to limit infiltration of the peripheral immune system into the brain. They respond to inflammatory cytokines and assist in the recruitment of peripheral circulating immune cells transducing signals from the brain parenchyma to the periphery and in reverse.

### 5.1. Differentiation Concepts

hPSC-derived models of BMEC-like cells (hiBMECs) have increased in popularity over the last decade, and consequently, a large number of potential differentiation protocols have emerged [20,147,148]. These protocols can largely be lumped into two categories, differentiations that produce cells with marginal endothelial phenotypes but display strong barrier properties and tight junction expression [25,149,150,151], and endothelial cells that have been differentiated through endothelial progenitor cells (EPCs), with the addition of small molecules to push the cells toward a BMEC-like fate, but lack substantial barrier properties [152,153]. There are efforts to attempt to bridge this gap, but a definitive BMEC differentiation has yet to be discovered [20,154]. Due to the differences between these models, it is particularly important to be mindful when selecting appropriate model systems [20].

However, existing models together offer excellent opportunities to study immune interactions and neuroinflammation within the neurovascular unit [155]. Thus far, however, most work with hiBMECs has primarily focused on transport, and the natural extension of disease modeling has begun to be explored [148,156,157,158].

### 5.2. Neuroinflammatory Insights

Early efforts with hiBMECs have shown that they have limited response inflammatory cytokines, though there are some conflicting reports depending on the model and application [159]. These findings have been summarized in Table 4. Barrier-forming BMECs following protocols based on the 2012 Lippman et al. paper show limited success in 2D but substantially more in 3D [160]. Using barrier-forming hiBMECs, it has been shown that hiBMECs increase uptake and transport of IgG proteins when inflamed and similarly when exposed to Aβ 1–42; interestingly, despite similar global results, the pathway for uptake was different between the two with caveolar transport being primarily responsible but micropinocytosis also playing a role in Aβ-mediated transport changes [85]. hiBMECs from a similar differentiation protocol show increased adhesion of PBMCs to the lumen of an artificial vessel stimulated with TNFα, but no change in permeability was shown when compared to a control vessel [161]. In the same model, it has been shown that TNFα increases the adhesion of monocyte-like cells in 3D, but not in 2D, and this effect is enhanced with pericyte co-culture [160]. Additionally, RNAseq revealed GO terms related to the cytokine-mediated signaling pathway (GO:0019221) and cellular response to cytokine stimulus (GO:0071345), which are enriched in 3D culture potentially partially explaining this differential response [160]. Another 3D culture based on the same model saw a significant dose-dependent increase in dextran permeability and retraction of astrocyte endfeet from vessels treated with inflammatory cytokines [80].

Barrier-forming hiBMECs in 2D have been shown to show elevated levels of ICAM-1 upon TNFα stimulation, but no expression of P- or E-selectin, and low expression of PECAM-1 [159]. This limited range of reactions to inflammatory cytokines requires additional models to be required for investigations on neuroinflammation. In differentiation protocols that go through an intermediary of an endothelial precursor, the inflammatory response is much more pronounced [159]. The endothelial precursor-derived cells have been shown to have robust increases in ICAM-1 expression, but no detectable expression of VCAM1 [153], and an increased expression of P-selectin following stimulation [159]. Similarly, when iPSCs generated from patients with multiple sclerosis are used to generate the BMECs, the increase in ICAM-1 expression following TNFα treatment increases further and VCAM-1 expression increases, and there is increased interaction with allogeneic Th1 cells [26]. Relatedly, hiBMECs have been used to screen anti-inflammatory compounds to treat LPS-primed PBMCs, and changes in IL-1β release are consistent with current successful therapeutics [164].

The use of hiBMECs to model neuroinflammation is just the beginning; it is clear, however, that careful selection of models is needed to collect accurate data. This work has also been predominantly performed in the spaces of AD and MS with other CNS diseases associated with neuroinflammation not currently studied.

## 6. Pericytes

Pericytes play a pivotal role within the neurovascular unit, actively contributing to the preservation of the BBB and regulation of cerebral blood flow [165]. They act as chemical sensors, enabling communication between the brain parenchyma and blood vessels to maintain optimal cerebral perfusion and brain function [165]. Pericytes contain contractile proteins such as desmin, vimentin, myosin, and nestin, enabling them to respond to neuronal activity and modulate cerebral blood flow [166,167]. Pericytes are involved in functions beyond facilitating cerebral blood flow. Recent studies have revealed their involvement in controlling the endothelial cell cycle and promoting basement membrane formation [168]. Mice lacking pericytes exhibited a reduction in microcirculation, leading to perfusion stress. Pericyte-deficient mice show disrupted BBB and increased neurotoxin accumulation and inflammation [169,170]. They have also been shown to play a critical role in neurodegenerative diseases such as ALS [171] and AD [172,173]. As the field continues to expand research into the many roles of pericytes, their roles in neuroinflammatory pathways are becoming more and more clear. Unlike the other cell types discussed in this manuscript, there are no clear examples of hPSC pericytes being used to study neuroinflammation. This field is poised for significant growth and discovery.

### 6.1. Role in Neuroinflammation

Pericytes play an important role in the inflammatory cascade within the brain, interacting with astrocytes, microglia, and peripheral immune cells, as well as the production of inflammatory molecules [174]. Pericytes express a variety of pattern recognition receptors (PRRs), such as Toll-like receptors (TLRs) and Nod-like receptors (NLRs), allowing them to recognize harmful stimuli and initiate inflammatory responses. Upon activation, pericytes release proinflammatory cytokines and chemokines and upregulate adhesion molecules to recruit immune cells [175]. They can also contribute to the formation of inflammasomes, which further amplify inflammatory responses [176]. Notably, pericytes have been shown to release IL-1β through non-canonical pathways, leading to pyroptosis [176].

Pericytes regulate neuroinflammation by influencing immune cell trafficking. They can upregulate adhesion molecules like ICAM-1 and VCAM-1, promoting neutrophil extravasation [177,178]. Moreover, pericytes can undergo morphological changes, facilitating macrophage infiltration into the CNS [179]. Additionally, pericytes respond to inflammatory stimuli by releasing proinflammatory cytokines and chemokines, such as IP-10 and ICP-10, and by altering their expression of proteins like TGF-β1 [180,181].

Pericytes are emerging as crucial mediators of neuroinflammation within the CNS. Their ability to modulate inflammatory responses and regulate blood–brain barrier permeability [182] makes them a key target for therapeutic interventions in neuroinflammatory diseases. The development of protocols to derive pericytes from hPSCs opens new avenues for studying neuroinflammation. These in vitro models can be used to investigate the mechanisms underlying pericyte-mediated neuroinflammation and to identify potential therapeutic targets.

### 6.2. Differentiation Concepts

Most of what we know about the role of pericytes within the cerebral vasculature comes from rodent in vivo data and limited studies using primary human pericytes. hPSC pericytes are in their infancy, and many protocols generating hPSC pericytes are focused on validating the phenotype, focusing on their effect on endothelial cells in stabilizing tube-forming assays or impacting permeability in a transwell model. These studies have been summarized in Table 5.

Recently, there has been a rapid increase in the number of hPSC-derived pericyte-like cells published. Several methods have been described for generating iPSC-derived pericytes, going through either mesenchymal or mesoderm lineages. These pericytes exhibit morphological features and express key markers, such as PDGFRβ, CD13, and NG2, characteristic of primary pericytes. Pericytes are present in organs other than the CNS, so to differentiate brain-specific pericytes, neural crest cells are first derived and then pushed to a mesenchymal-like fate using serum or other growth factor mixes [189]. Others use non-brain specific pericytes to give insight into the role of perivascular cells at the BBB [190,191,192].

Derived pericytes offer several advantages over primary pericyte lines. They can be derived from iPSCs reprogrammed from individuals with diverse genetic backgrounds and disease conditions, enabling their use in both fundamental research and disease modeling [193]. A recent study demonstrated the potential of pericytes derived from mouse embryonic cells to improve microcirculation in Alzheimer’s disease models [194]; the application of this technique to iPSC-derived pericytes from the same donor remains to be explored. Additionally, these pericytes can be co-cultured with other cell types derived from the same iPSC line, facilitating the creation of in vitro models of the neurovascular unit. Furthermore, their autologous nature makes hPSC pericytes potentially suitable for personalized medicine approaches.

One of the challenges in utilizing iPSC-derived pericytes lies in their characterization. The field lacks a definitive consensus on specific markers for brain pericytes, compounded by the known heterogeneity between pericytes associated with different vessel types: arterial, capillary, and venular [9,195]. This variability in pericyte phenotypes and the absence of universally accepted markers introduces complexities when employing these cells in modeling neurodegenerative diseases. The lack of standardized characterization can lead to inconsistencies in experimental results and interpretations. Despite this limitation, given the increasing recognition of pericytes’ role in neuroinflammation and the availability of protocols to differentiate hPSC-derived pericytes, these cells should be incorporated into in vitro and ex vivo models to further investigate this complex condition.

## 7. Conclusions

Neuroinflammation plays a significant role in various brain disorders, including neurodegenerative conditions, and methods to study inflammatory responses via hPSCs are of great importance. Their ability to differentiate into various neuronal and glial cell types provides the opportunity to model complex neuroinflammatory processes in vitro using increasingly complex models [196]. By leveraging hPSC technology, researchers can investigate the molecular mechanisms underlying neuroinflammation, identify novel therapeutic targets, and develop personalized treatment strategies.

hPSC-derived models offer several advantages over traditional animal models. They allow for the study of human-specific disease mechanisms, eliminating species-specific variations. Additionally, iPSCs can be generated from patients with specific neurological disorders, providing a unique opportunity to study disease-specific phenotypes.

The ability to isolate and study individual cell types, such as pericytes and OPCs, in vitro has significantly advanced our understanding of their role in neuroinflammation. While traditional in vivo models often provide a holistic view of disease processes, in vitro studies offer the advantage of focusing on specific cellular mechanisms without the confounding effects of other cell types or systemic factors. Recent research has highlighted the critical contributions of pericytes and OPCs to neuroinflammatory processes. By successfully differentiating and characterizing human pericytes and OPCs from iPSCs, researchers can gain deeper insights into human-specific disease mechanisms.

While hPSC technology holds immense promise, challenges remain. Refining differentiation protocols to generate mature and functional neurons and glia is crucial. Additionally, developing standardized methods for inducing and measuring neuroinflammation in hPSC-derived models is essential for reproducibility and comparison across studies. Working to overcome these challenges is key to realizing the potential of the FDA Modernization Act 2.0 of 2022 which has rolled back requirements of preclinical animal testing potentially increasing the role hiPSC-derived model systems may provide in bench-to-bedside where appropriate [197]. There are, however, limitations of hPSC-derived model systems, including unwanted variability influenced by the source of the cells, method of reprogramming, passage number, cell line, and cellular heterogeneity [198]. Cells derived from hPSCs are always imperfect models of the cell types they aim to replicate, sharing many of the same phenotypic characteristics, but lacking others [199]. Critical to the investigation of neuroinflammation in the context of aging, hPSC-derived cells are differentiated rapidly in vitro, often resulting in immature phenotypes. This inherently dictates the importance of verifying all results obtained using hPSC-derived cellular models. Appropriate methodological guidelines for increasing rigor and reproducibility specific to hPSC-derived model systems are established and well described by Volpato and Webber (2020) [200].

In conclusion, hPSC technology represents a significant advancement in the field of neuroinflammation research. By addressing the limitations of traditional models and providing a patient-specific approach, hPSCs have the potential to greatly enhance our understanding of neurodegenerative diseases and accelerate the development of effective therapies. This approach allows for the study of cellular and molecular changes associated with neuroinflammation associated with diseases such as Alzheimer’s disease, Parkinson’s, stroke, multiple sclerosis, and tumors, in a more controlled and precise manner.

## Figures and Tables

**Table 1:** Summary of the key findings from the use of hiMGs to study neuroinflammation.

Disease	Differentiation	Key Findings	Source
	Abud et al. 2017 [38]	hiPSC-derived microglia can phagocytize Stimuli of ADP triggers intracellular release of Ca	[58]
AD	Abud et al. 2017 [38]	Transcriptomics of hiMGs show similarities to human primary fetal and adult microglia hiMGs excrete cytokines and chemokines when exposed to proinflammatory molecules hiMGs phagocytize tau and Aβ	[38]
AD	Abud et al. 2017 [38]	Transcriptomics of hiMGs with APOE4 variants and KO reveal species-specific role of microglia in cholesterol metabolism in AD	[59]
AD	Muffat et al. 2016 [54]	Transcriptomics show that iMGs express APOE4 and a third of the genes are immune-mechanistic iMGs with less functional APOE4 phagocytize Aβ_42_ slower	[60]
AD	Gutbier et al. 2020 [61] adapted from Wilgenburg et al. 2013 [62]	TREM2-KO iPSC-derived microglia monoculture displays AD phenotypes including increased intracellular Ca release to ATP/ADP and increased complement C5a	[63]
AD	Transcription factor enhanced	8-day, 6-transcription-factor CRISPR iPSC → iMG differentiation Transcriptomics of iTF microglia show differences in microglial states associated with AD-associated response to stimuli including cytokine production and phagocytosis	[57]
PD	Wilgenburg et al. 2013 [62]	hiPSC-derived macrophages express aS at similar levels to patient-derived myeloid cells Increased expression of internal aS reduced phagocytosis	[27]

**Table 4:** Summary of key findings describing the neuroinflammatory role of hiBMECs.

Disease	Differentiation	Key Findings	Source
AD	Lippmann et al. 2014 [150]	Monoculture BMECs cultured with TNFα, IL-6, or Aβ Increased internalization and transport of IgG with stimulation	[85]
General Inflammation	Lippmann et al. 2014 [150]	Monoculture in 3D microvessel platform Treatment with TNFα increases PBMC adhesion	[161]
General Inflammation	Lippmann et al. 2014 [150]	3D microvessel culture required for TNFα activation Inflammatory phenotype increased with inclusion of pericytes RNA seq reveals 171 differentially regulated genes upon TNFα treatment	[160]
General Inflammation	Lippmann et al. 2014 [150]	3D-like structure including astrocyte co-culture TNFα, IL-1β, and IL-8 exposure result in decreased tight junction expression, increased permeability, and retraction of astrocyte endfeet	[80]
General Inflammation	Comparison between Lippmann et al. 2014 [150] Lian et al. 2014 [162]. Nishihara et al. 2021 [152]	BMECs show limited response to inflammatory cytokines EPC-derived BMEC show response more similar to standard ECs	[159]
MS	Nishihara et al. 2021 [152]	Cells derived from patients with MS show exaggerated inflammatory phenotype and enhanced interaction with Th1 cells and PBMCs	[26]
General Inflammation	Rosa et al. 2019 [163]	TNFα treatment resulted in increase in ICAM-1 and decrease in ICAM-2 with no detectable VCAM	[153]

**Table 5:** Summary of the key findings from hPSC brain pericyte differentiations.

Differentiation Progenitor	Key Findings	Source
Mesenchymal progenitor	Resulting pericytes aid in vascular tube formation in vitro and during integration in vivo Cells show both capillary and arteriolar-like phenotypes	[183]
Mesoderm/neural crest	Pericytes aid in vascular tube formation	[184]
Mesodermal lineage	Pericytes aid in angiogenesis	[185]
Neural crest	Pericytes mediate permeability transwell with BMECs Vascular self-assembly is observed	[186]
	Pericytes display contractile properties, promote neurological recovery, and rescue BBB function in a tMCAO model	[187]
	Contractile function and participation in blood vessel formation are disrupted in CADASIL	[188]

## Data Availability

Individual data can be found in the referenced manuscripts.

## Abbreviations

CNS	Central nervous system	FGF	Fibroblast growth factor
ROS	Reactive oxygen species	BMP	Bone morphogenetic proteins
RNS	Reactive nitrogen species	LIF	Leukemia inhibitory factor
GWAS	Genome-wide association studies	CNTF	Ciliary neurotrophic factor
HIF-1α	Hypoxia-inducible factor 1 alpha	GFAP	Glial fibrillary acidic protein
APOE	Apolipoprotein E	GLAST	Glutamate Aspartate Transporter
M-CSF	Macrophage colony-stimulating factor	AQP4	Aquaporin 4
hPSCs	Human pluripotent stem cells	Aβ	Amyloid beta
hiPCSs	Human induced pluripotent stem cells	TNF	Tissue necrosis factor
MS	Multiple sclerosis	NF-kB	Nuclear factor kappa light chain enhancer of activated B cell
PD	Parkinson’s Disease	COVID	Coronavirus disease
MHC	Major histocompatibility factor	NMO	Neuromyelitis optica
BBB	Blood–brain barrier	TBI	Traumatic brain injury
hiMGs	hiPSC-derived microglia-like cells	hiBMECs	hiPSC-derived brain microvascular endothelial cells
TMEM119	Transmembrane protein 119	VCAM	Vascular cell adhesion protein
P2RY12	Purinergic receptor P2Y G-protein coupled 12	JAK-STAT	Janus kinase signal transducer and activator of transcription
TREM2	Triggering receptor expressed on myeloid cell 2	TDP-43	Transactive response DNA binding protein 43
CD11b	Cluster of differentiation molecule 11b	OPCs	Oligodendrocyte precursor cells
CD45	Cluster of differentiation molecule 45	NG2	Neuron glia antigen-2
DAMPs	Damage-associated molecular patterns	hiOPCs	hiPSC-derived OPCs
PAMPs	Pathogen-associated molecular patterns	NPC	Neural precursor neuroectoderm-like cell
MAF bZIP	Musculoaponeurotic fibrosarcoma oncogene, basic leucine zipper	SOX10	SRY-box transcription factor 10
AD	Alzheimer’s disease	CLU	Clusterin
KO	Knockout	EPCs	Endothelial progenitor cells
ATP	Adenosine triphosphate	GO	Gene ontology
ADP	Adenosine diphosphate	ICAM	Intercellular Adhesion Molecule
aS	Alpha-synuclein	PECAM	Platelet Endothelial Adhesion Molecule
IL-1β	Interleukin 1-beta	LPS	Lipopolysaccharide
NLRP3	Nucleotide-binding domain leucine-rich-containing family pyrin domain-containing 3	PBMCs	Peripheral blood mononuclear cells
CRISPR	Clustered regularly interspaced short palindromic repeats	TLRs	Toll-like receptors
ALS	Amyotrophic lateral sclerosis	NLRs	Nod-like receptors
TBI	Traumatic brain injury	PRRs	Pattern recognition receptors
FTD	Frontotemporal dementia	IP-10	Interferon-inducible protein-10
hiA	hiPSC-astrocyte-like cell	TGF-β1	Transforming growth factor beta
		PDGRF β	Platelet-derived growth factor receptor beta
